# SSDraw: software for generating comparative protein secondary structure diagrams

**DOI:** 10.1101/2023.08.25.554905

**Published:** 2023-08-28

**Authors:** Ethan A. Chen, Lauren L. Porter

**Affiliations:** 1 National Center for Biotechnology Information, National Library of Medicine, National Institutes of Health, Bethesda, MD 20894; 2 Biochemistry and Biophysics Center, National Heart, Lung, and Blood Institute, National Institutes of Health, Bethesda, MD, 20892

## Abstract

The program SSDraw generates publication-quality protein secondary structure diagrams from three-dimensional protein structures. To depict relationships between secondary structure and other protein features, diagrams can be colored by conservation score, B-factor, or custom scoring. Diagrams of homologous proteins can be registered according to an input multiple sequence alignment. Linear visualization allows the user to stack registered diagrams, facilitating comparison of secondary structure and other properties among homologous proteins. SSDraw can be used to compare secondary structures of homologous proteins with both conserved and divergent folds. It can also generate one secondary structure diagram from an input protein structure of interest. The source code can be downloaded and run locally for rapid structure generation, while a Google Colab notebook allows for easy use.

## Introduction

Recent advancements in cryo-electron microscopy^1^, metagenomics^2; 3^, and deep learning-based protein structure prediction methods^4–7^ have led to an explosion in the number of available protein structures. For instance, the number of entries in the Protein Data Bank^8; 9^ (PDB), a repository of experimentally determined protein structures, has nearly doubled in the past 10 years. Earlier this year, the authors of ESMFold–a large language model that rapidly predicts three-dimensional protein structures from single sequences–released a web-based collection of >617 million predicted structures predicted from metagenomic sequences^6^. Similarly, >200 million structures predicted by AlphaFold2^4^–a highly accurate deep-learning based model for protein structure prediction–have been made available through a web repository for easy user access^10^, and many have been deposited into the UniProt database^11^ to model many protein sequences without experimentally determined structures.

The enormous increase in available models of protein structure presents opportunities to identify large-scale relationships between structure and properties such as sequence conservation or prediction confidence. Such relationships are often most effectively depicted when multiple protein structures are compared, motivating the development of structural alignment algorithms that match common elements of protein structure rather than amino acid sequence^12^. Nevertheless, important relationships between protein structures can be obscured by three-dimensional visualizations that cannot effectively convey all structural features through one image. This shortcoming especially impacts homologous proteins with non-conserved structural features arising from insertions, deletions, or mutations that cause substantial changes in secondary structure. Indeed, the need for easily interpretable comparative structure diagrams is underscored by several recent studies highlighting how protein structure can transform dramatically in response to seemingly minor sequence changes^13–17^. Comparative structure diagrams also simplify the visualization of fold-switching proteins, single sequences evolutionarily selected to remodel their secondary and tertiary structures in response to cellular stimuli^18–20^. In short, as increasing evidence indicates that highly similar or identical protein sequences can assume folds with drastically different secondary structures^21^, the need to graphically depict structural differences among homologous proteins and relate them to other protein properties increases.

To effectively depict relationships between the structures of homologous proteins and other properties of interest, we present SSDraw, a Python-based program that rapidly generates secondary structure diagrams from three-dimensional protein coordinates. These linear diagrams can be (1) registered using an input sequence alignment, (2) generated for multiple homologous sequences, (3) stacked for easy comparison, and (4) colored by any property of interest. These functionalities distinguish SSDraw images from other secondary structure visualizations^22–27^. For instance, ESPript^23^ relates secondary structures derived from one representative protein structure to multiple homologous sequences, usually divided on multiple lines of text. This format works well when the user seeks to visualize sequence conservation patterns in a protein family with conserved secondary structures. SSDraw may be preferable if the user seeks to compare structures of homologous proteins with divergent secondary structures by stacking each pre-aligned diagram and comparing structural differences. As another example, secondary structure diagrams from Aquaria^25^ also generate stackable linear secondary structure diagrams but color by sequence conservation only. SSDraw may be preferable if the user seeks to color the stacked diagrams by a property other than sequence conservation. In short, SSDraw was written to flexibly relate secondary structure differences between homologous proteins with other protein properties of interest. While this software was originally designed for fold-switching proteins^19^ and homologous sequences with different secondary structures^17^, it can also be used to generate single aligned or unaligned secondary structure diagrams for any desired use in seconds (local install) to minutes (Google Colab notebook).

## Results

### Software overview

**Figure 1.**
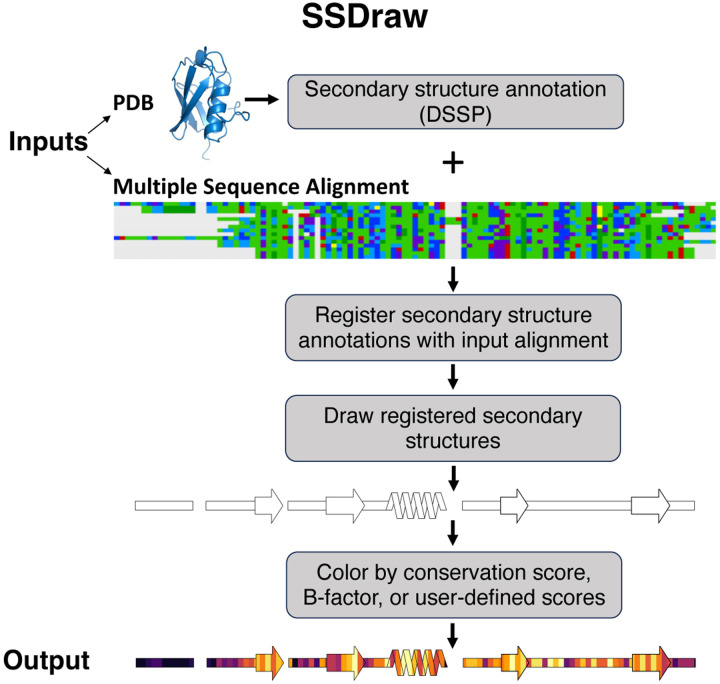
SSDraw flowchart. Inputs to SSDraw include three-dimensional coordinates of a protein structure in PDB format and a multiple sequence alignment in FASTA format. Protein secondary structure is determined from the input PDB using DSSP. Stacked polygons represent α-helices, arrows represent β-sheets, rectangles represent coils, and gaps are empty spaces. Secondary structures are registered with the input alignment to account for gaps and drawn using the Matplotlib patches library for Python3. Finally, secondary structures are colored by sequence conservation scores, B-factor, or another user-defined input. In this figure, the final diagram is colored by sequence conservation scores. Output structures can be saved as .png, .eps, or .tiff files at a user-specified resolution. Multiple sequence alignment depicted using the Alignment Viewer program: https://github.com/sanderlab/alignmentviewer.

SSDraw requires two inputs to run: (1) a file containing three-dimensional protein coordinates in PDB format and (2) a multiple sequence alignment in FASTA format (Figure 1). If a continuous nonaligned diagram is desired, the user may input a single ungapped FASTA sequence. The user may also specify the chain ID if they input a multi-chain PDB. SSDraw requires only alpha carbon coordinates to generate an image. The multiple sequence alignment can be generated with programs such as MUSCLE^28^, Clustal Omega^29^, or HMMER^30^, so long as it is inputted in FASTA format.

By default, SSDraw computes secondary structure annotations for each amino acid using Define Secondary Structure of Proteins (DSSP)^31; 32^, which annotates secondary structure from three-dimensional protein structures based on hydrogen bonding patterns (Methods). In lieu of a PDB file, users may input alternative secondary structure annotations^33^ or pre-computed DSSP annotations in DSSP or .horiz format.

Annotated secondary structures are then aligned in register with the input sequence alignment (Figure 1) in FASTA format. For proper alignment, the user inputs the name of the reference sequence in the alignment. Lengths of the reference sequence and secondary structure annotations need not be identical; SSDraw will adjust the reference sequence to be the same length as the secondary structure annotations.

Secondary structures are then drawn with patches from the Matplotlib^34^ package for Python3 (Figure 1, Methods). Successive slanted polygons are used to represent α-helices, arrows represent β-sheets, rectangles represent loops, and empty spaces between secondary structures represent alignment gaps. Loops are layered under secondary structures. Segments of regular secondary structure shorter than 4/3 successive residues (α-helices/β-sheets) are represented as loops, as are β-turns (Methods).

If desired, secondary structures can be colored by sequence conservation score, B-factor, or another user-defined input (Figure 1). This feature was originally developed to compare secondary structure conservation in a family of bacterial response regulators with some secondary structure elements that switch from α-helix to β-sheet in response to stepwise mutation^17^. The coloring implementation for these figures was slow because each individual secondary structure element was colored successively (i.e. each polygon in each helix) by masking a colormap corresponding to user input scores. For SSDraw, we improved performance ~22-fold by coloring all identical secondary structure elements together, leading to 4 coloring steps (one for all β-sheets, one for all loops, and two for all a-helices) rather than dozens or hundreds (N_1_ for each polygon in each α-helix, N_2_ for each β-sheet, and N_3_ for each loop segment, Methods). Sequence conservation scores are computed automatically from the input sequence alignment (Method). Alternatively, the image can be colored with a solid fill specified by the user. For instance, the first diagram in Figure 1 was generated using a white fill. Custom coloring schemes and custom colormaps may be specified by the user.

If the user wants to assign custom coloring scores to each residue, they have two options. The first is to upload a custom scoring file that contains residue-specific scores. This file is formatted with two columns: column one contains one-letter amino acid codes for each residue to be colored; column two contains scores corresponding to the amino acids in column one; columns are delimited by one space. The second option for custom scoring is to input a PDB file with C-alpha B-factors corresponding to custom scores and coloring the image by B-factor. This option allows the user to easily visualize confidence scores from structure predictors such as AlphaFold2^4^ and ESMfold^6^, if desired. Any range of scores can be used for custom coloring: scores are normalized before the image is colored. Because SSDraw uses the Matplotlib^34^ Python package, any premade Matplotlib colormap may be used; users can also specify custom colormaps as input.

For those desiring to visualize a protein region rather than a whole region, starting and ending residues can be specified. The Google Colab notebook provides a sliding window that allows the user to select which portion of the alignment will be drawn. Residue numbers corresponding to PDB numbering can be inputted into the local install.

The final output is a linear secondary structure diagram, colored as the user specifies (Figure 1). Output files can be saved as .png, .eps, and .tiff files at a user-specified resolution. By default, figures are saved as .png files at 600 ppi (pixels per inch), a publication-quality resolution.

### Making comparative secondary structure diagrams

**Figure 2.**
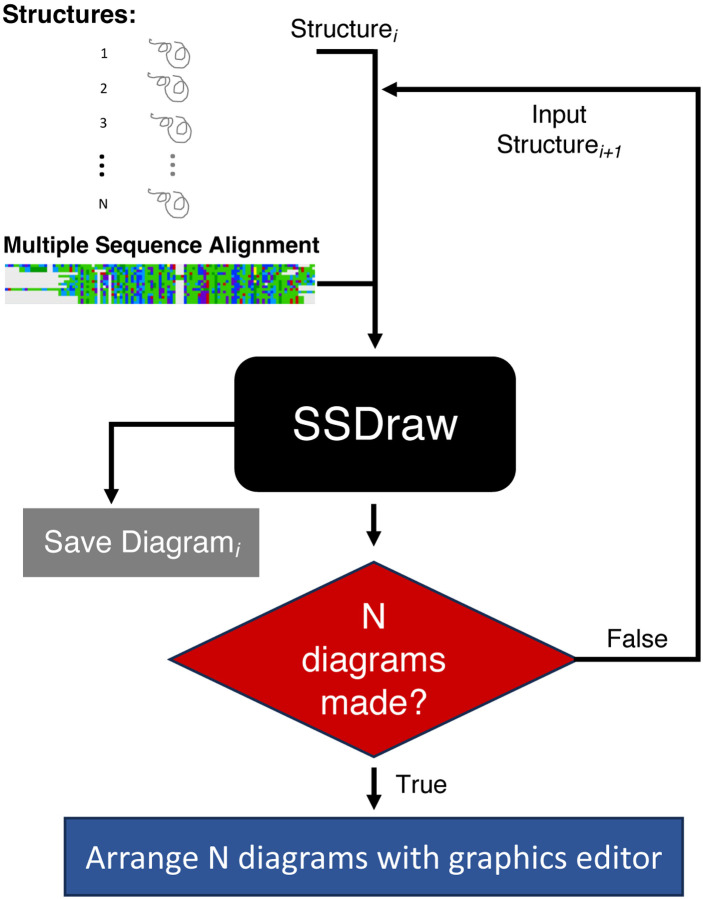
Generating multiple secondary structure diagrams with SSDraw. Three-dimensional coordinates corresponding to each of N protein structures are inputted into SSDraw one at a time along with a multiple sequence alignment (MSA) that registers the secondary structure annotations of all N structures. During run i, Structurei is inputted into SSDraw along with the secondary-structure-registering MSA. SSDraw will output secondary structure Diagrami. This process is repeated for all N protein structures. The same MSA is inputted for each run. After all N diagrams are generated, a graphics editor–such as Adobe Illustrator or Microsoft PowerPoint–can be used to orient them as desired.

One of SSDraw’s main utilities is generating multiple secondary structure diagrams that can be stacked and compared. Figure 2 depicts how this can be done. PDB structures are inputted individually into SSDraw along with an MSA in which the sequences of all desired diagrams are aligned. It is important to note that the same MSA is inputted into each run to ensure that each diagram is registered against the same alignment. This allows for consistent comparison between all diagrams. Consequently, only the input PDB file changes with each run, unless the user requires custom input files such as coloring scores for each input PDB. For users running SSDraw locally, we recommend writing shell scripts with sequences of commands to generate each diagram successively. After all individual structures and their reference MSA is inputted, a secondary structure diagram will have been generated for each structure. These diagrams can then be oriented as desired by using a graphics editor such as Adobe Illustrator or Microsoft PowerPoint. Automatic diagram orientation is not included as an option with SSDraw. In our experience, custom orientation and labeling usually yields the best results.

### Examples

#### Comparing distinct structures with highly identical sequences using a custom color map

**Figure 3.**
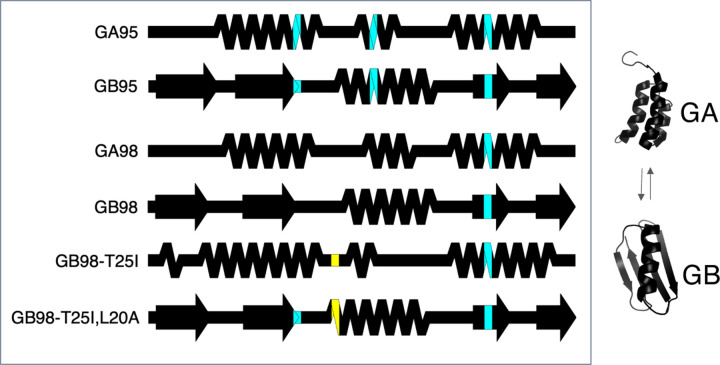
Comparing the structures of proteins with highly identical amino acid sequences but different folds. Diagrams show secondary structures derived from the nuclear magnetic resonance structures of engineered variants of human serum albumin binding protein GA (trihelical bundle) and immunoglobulin binding protein GB (4b+α fold). Pairwise sequence identities range from 95% and 98% (labeled with 95 and 98, respectively). Positions that switch the folds are colored from top to bottom. Thus, position 20 of GA/GB 95 are colored because it switches their folds, but it is black from GA98 because the position was not mutated from GB95 directly above. Positions 20, 30, and 45, where fold-switching mutations have been identified, are cyan. Mutations in these three positions foster fold switching in both between GA/GB95 and GA/GB98 variants. Position 25, in which mutations have been found to flip folds in GA/GB98 variants only is yellow. Three-dimensional structures of GA and GB folds (PDB IDs 2KDL and 2KDM, respectively) shown to the right.

SSDraw can be used to compare secondary structures of proteins with high levels of sequence identity but different folds (Figure 3). Extensive work has been performed to engineer^15; 35–37^ and characterize^16; 38; 39^ variants of the human serum albumin-binding protein GA and the immunoglobulin binding protein GB. While GA folds into a trihelical bundle, GB folds into a 4β+α structure. One or several mutations can cause the protein to flip from one ground-state fold to the other^36; 37^. The distinct secondary structures of GA and GB variants can be visualized readily with SSDraw. In Figure 3, identical residues are colored black while residue positions with mutations found to foster fold switching are cyan and yellow. Mutations in cyan positions foster fold switching in GA/GB variants with both 95% and 98% sequence identities, while mutations in yellow positions have been observed to flip folds in GA/GB variants with 98% sequence identity only. Black amino acids in 98% identical variants that correspond to cyan positions in 95% identical variants signify no amino acid change from GB95. Coloring the diagrams in this manner shows that most fold-switching mutations among these variants occur in secondary structure rather than loops. Furthermore, fold-switching mutations tend to occur in the central region of the protein (residues 20, 25 and 30) rather than at the termini, where the closest known fold-switching mutation is 11 residues away from the C-terminus (position 45).

#### Comparing sequence conservation in similar structures with a default color map

**Figure 4.**
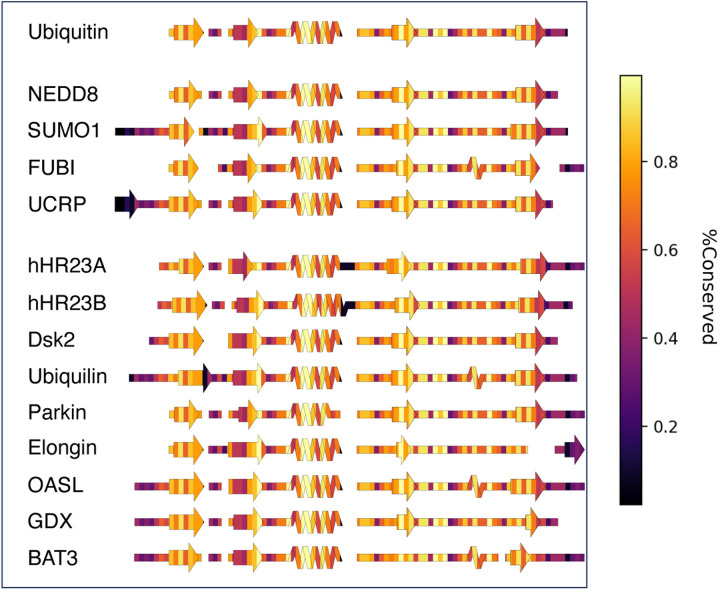
SSDraw diagrams for ubiquitin and ubiquitin-like proteins colored by conservation score (1.0 is highly conserved; 0.0 is least conserved).

SSDraw can also be used to relate sequence conservation to secondary structure in protein families with conserved folds. These comparisons for ubiquitin and ubiquitin-like proteins^40^ are shown in Figure 4. Not surprisingly, sequences in loop regions tend to be least conserved, while sequences that fold into secondary structures tend to be more conserved.

One exception is the second beta sheet, which has been identified as a SUMO1 and putative NEDD8 binding motif by NMR spectroscopy^41^ and structural modeling^42^, respectively. Thus, sequence variation in the second β-sheet may foster different binding functions in different ubiquitin-like proteins. Sequence conservation was calculated directly from the input sequence alignment (Methods).

## Discussion and Conclusions

SSDraw generates publication-quality secondary structure diagrams in seconds to minutes. These diagrams can be colored by conservation score, B-factor scores, or a user-specified metric, allowing relationships between secondary structure and other protein properties to be observed readily. SSDraw is expected to be most useful for comparing secondary structures of homologous proteins with different folds, an emerging class of proteins^43^ for which few computational tools are available. Nevertheless, SSDraw may also be used to (1) diagram single structures and color them by any property of interest and (2) compare secondary structures of homologous proteins with conserved folds.

## Methods

### Secondary structure annotation

SSDraw uses DSSP^31; 32^ to annotate secondary structure from three-dimensional protein coordinates in PDB format. The local install uses the DSSP module in Biopython^44^ to parse the annotation generated by separate compiled software. Only C-alpha coordinates are necessary for annotation. In addition to regular secondary structure (α-helices and β-sheets), DSSP annotates various local structures such as β-turns and 3_10_ helices. These features are not displayed in SSDraw diagrams. Helices are drawn for at least 4 consecutive “H” annotations, and β-sheets are drawn for at least 3 consecutive “E” or “B” annotations, combined in any way. All other annotations are visualized as loops. Short helices with <4 consecutive “H” annotations and short β-sheets with <3 “E” or “B” annotations are also visualized as loops.

### Drawing secondary structures

Annotated secondary structures are grouped into three categories: Loop, Helix, and Strand. The lengths of each segment of structure in each category are recorded. Then, each category is drawn separately using the patches library from Matplotlib^34^ for Python3. First, Loops are drawn. Loop lengths are calculated as the number of consecutive annotations divided by 6.0 with the Rectangle patch. When Loops connect elements of secondary structure, they are extended at both ends by 1.0/6.0. All loops have a zorder of 0 so that their images are layered under strand and helix diagrams. Then, coordinates for images of β-sheets and α-helices are stored to be drawn later for better performance. Strands are drawn using the FancyArrow patch with a width of 1.0, linewidth of 0.5, zorder=index increasing over all secondary structures from left to right, head_width of 2.0, and head length of 2.0/6.0. Length is defined as the number of consecutive annotations for the strand being drawn/6.0; to avoid incorrect gapping, this length is extended by 1.0/6.0 if C-terminal elements of secondary structure follow the strand. Helices are drawn as stacked Polygon patches with right-leaning patches layered on top and left-leaning patches layered underneath. The short sides of the polygons measure 1.0/6.0; the long sides measure 1.8/6. Helices begin and end with shorter polygons that align with other secondary structures (height of 1.4/6, width of 1.0/6). All lengths are proportional measures scaled to fit into a figure 25 inches long. Consequently, shorter proteins will have larger secondary structures in the horizontal dimension and vice versa. Vertical heights of all secondary structures are kept constant. Loops less than three consecutive residues between two gaps are not drawn.

### Coloring secondary structures

Secondary structures have black edges; their insides are filled by clipping an input colormap equal in size to the diagram. Groups of loops, helices, and strands are each converted to clipping paths using Matplotlib’s mpath.Path command. These paths are then converted to patches with mpatch.PathPatch. Finally, an input colormap equal in size to the diagram is generated from user specified parameters or a solid color and clipped to fill the insides of the path (im.set_clip_path command); the rest of the colormap is discarded. Repetitively generating the colormap slows performance considerably. For instance, generating one diagram of a 215-residue response regulator with a mixture of helices and strands (PDB ID: 1A04) takes 1 minute, 5 seconds when a colormap for each secondary structure element–including every polygon to make the helix–must be generated. To improve performance, SSDraw generates colormaps 4 times—once for loops and β-sheets and twice for a-helices: once for the bottom left-leaning layers, and once for the top right-leaning layers. Running this improved implementation hastened image generation of 1A04 to 3 seconds, a ~22-fold speed-up from 1 minute, 5 seconds. The Google Colab notebook takes about 2 minutes to generate its first secondary structure diagram because it must load outside software packages, such as DSSP, before running.

### Conservation Scores

Conservation scores are computed directly from an input sequence alignment. First the consensus sequence is determined by calculating the most common amino acids in each column of the alignment. A conservation score is then calculated by:

1. Determining the number, *N*, of amino acids in column *i* with substitution scores ≥ 0 for the consensus residue in column *i*. Substitution scores are calculated using the BLOSUM62^45^ matrix supplied by Biopython^44^.
2. *N* is then normalized by the total number of amino acids in column *i*. Gaps are not included in the normalization.
